# Foreign Summary

**Published:** 1840-05-16

**Authors:** 


### FOREIGN SUMMARY.

### PHILLIPS' LECTURE ON THE PRINCIPLES AND PRACTICE OF SURGERY.—NO. V.

Wounds—(Continued.)

Incised *Wounds: Treatment.*

Contused *and* Lacerated *Wounds: Peculiarities—Treatment.*

Gun-shot *Wounds: Their Characters—Varieties—Symptoms.*

*Treatment.*—In the treatment of incised wounds, the principles are simple and obvious; if the wound be not complicated by the presence of a foreign body, bring the edges together, and keep them so; and if any accidents arise, treat them. To accomplish these indications, certain things are necessary, a good position, adhesive straps, bandages, sutures. First, with regard to *position*, you would not place a man on his back who had a wound of the occiput, and, *vice versâ*, if a wound implicated the extensor muscles of the limb, you will place the limb in extension; the ends of the divided muscles will then be brought nearest together. If it implicate the flexor muscles, flexion will manifestly be the position. Supposing you have to treat a wound implicating, transversely, the anterior parietes of the abdomen, you would not place a pillow in the small of the back, but you would raise the pelvis and the chest. If the wound were longitudinal, implicating the fibres of the rectus, an opposite course would be necessary. Again, position is of great importance with reference to the circulation, to the reflux of venous blood. If the leg be wounded, and allowed to hang down, the course of the arterial blood towards the point will be facilitated; that of venous blood from the part will be interrupted by the influence of gravitation. So decided is this influence, that Piorry stated, what I have partially confirmed in one case, that hæmorrhage from the radial or ulnar artery may be arrested by keeping the arm up above the head. We must, however, be careful, that in attempting to get rid of one inconvenience we do not incur another; that, in attempting to facilitate the return of venous blood, we do not prevent the escape of pus.

With regard to *strapping*, that of isinglass I prefer, as being less irritating. Straps act by their adhesion to the integuments; therefore their force is, to a considerable extent, dependent upon the adhesiveness; to a great extent upon their length: thus a strap two inches long will act with much more force than a strap of one inch. Straps act only on the integuments; therefore, when we wish to act on deeper parts, we must employ some other agent. There are, it is true, certain muscles which may be acted upon by the skin, the platysma, the occipito-fontalis, the orbicularis oris—still the moisture about the lips will loosen the straps, and sutures may also be necessary.

By means of *bandages*, we can, in certain cases, exert more power in bringing parts together than by adhesive straps. They act more decidedly on the deeper seated parts; they are especially convenient in longitudinal wounds. The bandages commonly employed for this purpose are either tail bandages or rollers. It is often necessary to associate compresses with the bandage. Supposing we are dressing a longitudinal wound, affecting the anterior part of the thigh, you would place one compress to increase the support at the inner, and one at the outer side of the wound. But even when well applied, it is seldom that the bandage is perfectly efficacious, that it exactly maintains contact between the edges of the wound ; much of its action is lost in compressing the part perpendicularly to the surface. Still it prevents the wound from gaping as much as it might otherwise do. Compresses well applied have this advantage, that they only make pressure at two points of the circumference, and therefore interfere less with the circulation than circular bandages.

Of all the means employed to maintain wounded surfaces in contact, *sutures* are undoubtedly the most efficient. The older surgeons employed them perhaps too frequently. The moderns came to the conclusion in the last century, that so many inconveniences attached to the use of the suture, that it should be very rarely used, and by some it was actually proscribed. Within a few years, a new doctrine has been promulgated, and it is recommended in a large number of cases. Still, differences of opinion exist with regard to it at present. The introduction of two or three needles cannot be done without pain; it is also said that they irritate the muscles, and produce a tendency to spasmodic contraction. A still graver reproach has been made to them, that they break through the tissues, and are thus more hurtful than useful. It is very true that this occasionally happens, but from imprudence, by endeavouring to overcome a resistance which was not likely to yield. Those of you who attend hospital practice have seen their great efficacy in hare-lip, wounds of the face and abdomen, and elsewhere when too powerful muscles are not implicated ; but if you attempt with threads to bring together large wounds of the deltoid or the muscles of the thigh, you may expect to experience the inconveniences we have spoken of. They will be found useful where a flap exists with its base downwards, and where the flap must be sustained. Such as a sword cut in the deltoid. Here there is no resistance to overcome ; all that is required is support. It will be found very useful in wounds occurring in elderly people, who have been very stout, but are no longer so. In them the skin is very flaccid, the surfaces can be easily brought together, but the flap tends to curl up, and union is prevented. Sometimes there are indications for using the suture more pressing than the fear of deformity ; the masseter and buccinator muscles are cut through ; the parotid duct is implicated ; if union does not take place, fistula will succeed. In wounds of the eyelid, it must often be employed. There are parts which are occasionally divided, and where position, strapping, bandaging, will not succeed—the tongue, for instance ; here the suture can alone be used. Again, it is no less necessary in wounds of the abdominal cavity, out of which the viscera may glide, if the wound be large enough to allow of their escape. Still, the use of the suture in large abdominal wounds is sometimes followed by convulsions, vomiting, intense pain, which resist every kind of treatment ; therefore they should not be used unless the necessity be absolute. Some men use the suture very generally, because contact is more exactly and more certainly maintained, and the necessity for firm bandaging, from which, in many cases, congestion at the wounded surface results, is done away with.

Sutures are not all of one kind : those at present commonly employed are the interrupted suture, the twisted, and the quill suture. The first is made by passing a needle armed with a thread, from without inwards of one lip of the wound, and pressing it from within outwards of the opposite side, and tying a knot on the surface. Where there is not much resistance, this does very well, as in sustaining a flap for instance, in amputations of the breast, the testicle, &c. If the object of the suture be to maintain very exact contact, this suture will not do. In any autoplastic operation, you do not see it employed, but the twisted suture. In the twisted suture the needles remain ; we insert them as much deeper, as it is possible the resistance will be greater. Having inserted the needles, having taken care that they have penetrated at exactly opposite points, the lips are brought together by twisting the suture either circularly or in the form of a figure of 8. In this way more exact coaptation is maintained. These needles are left in place for three or four days ; at that time union is usually sufficiently advanced to admit of the removal of one or all, according as union may be more advanced at one point than another. If the needles destroy the tissue, it is usually from the inattention of the surgeon : if there be likelihood of its giving way, much may be done by supporting them with straps or bandages. The quill suture is made by passing threads through both lips of the wound as in the interrupted suture, but instead of knotting them upon the surface, we fix them on each side upon a cylinder of bougie or quill, or other convenient substance. The advantages of this kind of suture are, that the drag, instead of being upon several points, which may give way, is spread over a considerable surface, and we may thus employ with impunity a greater force for bringing the parts together ; more action is also exercised upon the deeper parts of a wound. The quill suture is therefore the best means of procuring union in a wound, where there is considerable fear of the parts being dragged asunder—certain wounds of the abdomen, lacerations of the perineum. The use of different sutures may thus be summed up. The interrupted suture does very well where the wound is superficial, where much force is not required, and where very exact adaptation is not important : the twisted suture, when exact adaptation is necessary ; the quill suture where there is much resistance, where the wound is deep, and where all parts of it should equally be brought together.

In the treatment of wounds, however, very often more is necessary than position, strapping, bandages, and sutures. We seek to obtain union by "first intention;" if the inflammatory action be insufficient, or if it be more than sufficient, this effect may not be obtained; and therefore it is most important to excite it when insufficient, to moderate it when excessive. If the inflammatory action were first sufficient, it would be obviously improper to employ, on the one hand, irritating applications, and on the other relaxing ones. In most cases cold water is a good application; but if energetic local depression of vital action be required, irrigation will be found more effective. This irrigation may be accomplished conveniently by suspending a bottle of cold water in a proper position; placing in it a few threads of lamp cotton, one extremity of which should reach to the bottom of the bottle, the other hang out at its mouth: in this way you get a species of syphon, and a constant dropping upon the lint or linen covering the part. The part should, of course, be kept at rest. The mind should also be kept quiet; mental disquietude often exercises a very decided influence upon the healing of wounds. It is, probably, because the mind is quiet, that, in the operation of lithotomy, nine children out of ten do well; it is probably why, in brute animals, wounds generally do well. Certain persons are more irritable or excitable than others, and in those wounds often do ill. Thus, in wounds of the head, it is important that the eyes should not receive the impression of light. In bad wounds, noise is often prejudicial; thunder, or a discharge of fire-arms, has been known to excite tetanus. Moral and physical quiet are, in many wounds, most important.

With respect to food, I think the common practice incorrect. I rarely diet rigidly after operations; I think it is often injurious. I feed carefully until traumatic fever is developed; I then lessen the quantity of food until that subsides, then recommence the proper diet. The ordinary treatment is different: the diet is very rigid until free suppuration takes place. I support the opposite opinion, because I have seen the bad effects of abstinence after great operations or wounds. My plan is, to let the patient's diet be much what it was before the injury, even though it might be objectionable; and this I do upon the Hippocratic principle, that bad things, when habitually taken, often do better than good things to which the patient is unaccustomed. When a patient is suddenly subjected to rigid diet, the biliary secretion continues to be poured into the intestine, and in many cases irritates it. I would not wish you to stuff your patients, but I wish you to feed them moderately. There are patients affected with worms; if you place them upon a rigid diet, great intestinal discomfort will be experienced: give the patients food, and these discomforts will be dissipated. In hospitals it is especially necessary to direct attention to these things, because your orders are more clearly carried out than in private families; and I believe it is because such orders are not strictly carried out that wounds often do better at home than in hospitals. Do not, therefore, ride the abstinence hobby too hard; let your patients have a moderate quantity of digestible food.

It is very important to attend to the state of the bowels: when a patient is constipated, the abdominal circulation is troubled, the general circulation is accelerated, and there is often headach. It is never well, in large wounds, that there should be much effort to go to stool. A surgeon of great eminence, not many years ago, operated for stone: after the operation, he did not inquire as to the state of the bowels; for ten days all went on well. The patient strained in endeavouring to go to stool, burst a blood-vessel, and died of hæmorrhage. Quiet and regimen—are they always sufficient in all wounds? Must we sometimes bleed? If the patient be strong, but has lost much blood, the face be pale, and the loss seem sufficient to prevent febrile reaction, it is clear that we should not further deplete. It is the same in a debilitated constitution, even when little blood is lost. In both cases bleeding would be injurious. If, however, the patient be in rude health; has lost little blood; has a large, deep wound; a hard, quick pulse; a heavy head; a suffused countenance; the indication is manifest: to spare blood-letting would be a fault; the quantity of blood circulating in his veins should be diminished.

An incised wound may not heal by first intention; it may suppurate: when we remove the dressings by which we had hoped immediate union might have been obtained, the wound is gaping, and a puriform fluid may be seen; sutures, if any, may be at once removed, or they will give way; if, however, it be a great object to keep the edges, as nearly as may be, approximated, they may be left unless they excite irritation. In these cases the edges will never be brought to touch each other, but granulations are developed; a tegumentary membrane ultimately covers them; it thickens, becomes redder, but ultimately acquires a whiter colour than the adjoining tissues. This membrane is at first slowly formed along the edges, more rapidly as it approaches the centre, and appears to drag the edges of the wound towards the centre, so as to lessen the surface. This tissue never acquires the character of the cutaneous integument, has no papillæ, and is a cellulo-fibrous and vascular tissue.

Tactile sensibility is less remarkable than in the skin. Sometimes, either from negligent dressing or other cause, cicatrization is badly done; the surface is puckered; these cicatrices are not only disagreeable to look at, but often they interfere with the motion of parts: these are seen most frequently in burns.

In all cases, the present system, in my opinion, is, to interfere too much, to dress too often; when a wound is dressed, it should be left for many days; until, in fact, the dressings are very wet with the fluid secreted by the wound, or very offensive, or pain or uneasiness be felt. I am quite sure that the less frequently, consistently with the circumstances I have mentioned, dressings are renewed, the sooner a wound heals.

*Contused and lacerated Wounds.*—A lacerated wound may be produced by the bite of a horse or a cow, or any similar animal, by the action of a wheel passing over a part, by the action of machinery, or by any similar agency.

If an articulation be implicated, the ligaments seem to give way first, the skin and muscles later: we can scarcely say the muscular fibres are ruptured; they are separated from the tendons, or the periosteum. In Mr. Langstaff's museum are the ends of two thumbs which have been torn off, with a long tendon attached to each, but no muscular fibres. Usually, however large be the blood vessels ruptured, hæmorrhage ceases spontaneously; that is owing to two circumstances: the vessels are almost always ruptured high in the substance of the mass, so that they are compressed on all sides, and the three arterial tunics being unequally extensible, are ruptured at different levels; the internal and middle give way first, and are therefore ruptured highest; the external, more extensible, bears considerable elongation before it gives way, and when it yields, forms a kind of cone, which is ruptured much lower than the others; it is retracted, twisted like a cork-screw, and effusion of blood is prevented. Usually these wounds are not very painful, and the consequences are often not serious. It rarely happens that the pain is very acute; the patient is usually calm, and tetanus is very rare; suppuration occurs early, and cicatrization soon follows. Many remarkable cases are on record, none more so perhaps than that of Cheselden; the patient was a miller, who had around his arm a rope which got entangled in the wheel of a mill, which was revolving rapidly; he was raised from the ground until his body was arrested by a beam which prevented further ascent; the wheel continued to revolve, and the arm and scapula were separated from the rest of the body. He was unaware of the extent of his injuries until he saw his arm moving around on the wheel; there was no bleeding, he was able to walk down a ladder, and walk some steps to look for assistance. He got well in two months. We shall now proceed to consider contused wounds, which are often very similar to those on which we have been speaking.

*Contused Wounds.*—Contused Wounds are produced by blunt bodies moved with more or less velocity; they participate of the nature of contusions and of ordinary wounds. The contusion is sometimes superficial, limited to the skin and subcutaneous cellular tissue; it may be more intense, and implicate the whole thickness of a limb. Sometimes it appears trifling upon the surface, while it is severe underneath; in these cases the superficial parts have yielded, while the deeper-seated parts have resisted. A rounded body strikes the abdomen obliquely; there is no trace of contusion on the skin; even the muscles are only slightly contused; yet there may be rupture of the aorta, the vena cava, the liver, or the intestines, and sudden death. When the contusion is slight it leaves few traces, and scarcely any pain; when severe, there is ecchymosis, pain, tumefaction. If you dissect a recently contused part, when you cut into the cellular tissue it is more or less gorged with blood; in some cases the blood is still liquid, many vessels are ruptured; if you carefully examine you find a certain quantity of laceration. Upon the surface the discolouration is soon seen, and it terminates in a yellowish areola; these clots of blood are not always absorbed, sometimes they break down, become painful, and require operation for their removal. The quantity which may be absorbed is sometimes enormous. When the contusion is accompanied by a wound it may be regular, and the edges may be very little ecchymosed; they may be jagged, infiltrated with blood; and if it be narrow there may be extravasation of blood in the thickness of the parts. All contused wounds are not painful; but if the contusion be severe there may be absolute insensibility of the part; it may produce a state very like the death of the part. Lamotte describes a case where a billiard boy was struck on the arm with a cue, so severely, that his arm seemed dead for ten days; it was only at the end of that time that heat and sensibility returned to the part.

In a contused wound it is sometimes difficult to determine whether the tumefaction depends upon inflammatory action or extravasation; generally the extravasation of blood is immediate, or nearly so; the inflammatory tumefaction is not usually much developed for twenty to forty hours.

In some cases the edges of a contused wound will heal without suppuration, but it is not usually the case. In some instances the inflammation is so violent as to destroy the patient before gangrene is developed; in others, the suppuration is excessive, and the patient dies exhausted.

*Treatment.*—The important question to be considered is, how are these wounds to be treated; should we attempt union by "first intention?" No general direction can be given on this subject; nothing is more variable than such wounds; a part of such a wound may heal by "first intention;" another part will suppurate; this usually depends upon the degree of contusion. As these wounds, when left to themselves, almost always suppurate, it is desirable in many cases to seek to prevent, in others to confine it within narrow limits. If simple, it should be carefully cleaned with a fine sponge and warm water, to remove coagula, or any other foreign substance which may adhere to it; then bring it together with strapping or suture. If you can bring together parts whose contact will not irritate, you will do better than by interposing lint or any similar substance. Depend upon it, a flap is, for the part from which it has been detached, the best topical application that can be made. Supposing the parts to be so contused that union is almost impossible, still you may bring the parts together, and gently support them with a bandage; but it should be carefully watched.

In France, Pelletan used to stuff these wounds with "charpie;" Dupuytren brought them together partially; Boyer completely. In these cases simple cold water irrigations are often very beneficial. Undoubtedly one of the best means of preventing inflammatory accidents in severe cases is general bleeding; leeches in the vicinity of such wounds I do not like; they frequently seem to increase inflammatory action, and often occasion erysipelas. In these wounds, spite of bleeding, rest, careful dieting, and attention to the bowels, gangrene will sometimes occur: in such cases warm emollient poultices will be useful; tonics to the wound and internally are, in cases of gangrene in contused wounds, usually improper. If it be limited to the integument, the sloughs will come away in due course. These sloughs are not always dark. If the contusion be very severe you should not think of amputation until all hope is lost; first, because we should always endeavour to avoid mutilation; second, because these amputatious are often fatal.

Surgeons of great experience have been in the habit of applying aromatic and spirituous substances to contused wounds, but now they are abandoned. At present men's minds seem to be divided between emollients, narcotics, and sedatives, but cold water is in most cases preferable to either; the necessary means should have been taken for bringing together and dressing, before the cold pads are applied.

*Gun-shot Wounds.*—Although as civilians few of us can have had much opportunity of seeing gun-shot wounds, they are held to form a necessary part of a course of Lectures on Surgery. We must, therefore, conform to the ordinary arrangement, by giving a condensed account of these injuries. The only opportunity which I have had of observing these wounds, was at Paris, in 1830, and although that experience would be insufficient to justify me in founding a consideration of these injuries upon those events, yet it affords me a means of testing the views of others, and selecting those which experience convinces me to be the soundest views on the subject. This class of inquiries found little consideration in surgical works before the 15th century, but even in the 16th they were regarded as poisoned wounds, and also as burns. In accordance with these opinions the two indications presented were, to cure the burn and to destroy the venom. Sprengel states his belief that to Paré and to Maggi must be attributed the change in opinion as to the burn and the poison.

Under the term gun-shot wounds we include all wounds caused by projectiles propelled by gunpowder. A gun-shot wound may perforate the integuments at one or two points; that is, the ball may have remained in the body, or escaped. Where there are two openings they may be directly opposite one to another, or they may have no apparent correspondence with each other. The direction of the ball, when losing its velocity, may be changed by many objects—by a bone, a tendon, or even an aponeurosis. A cavalry officer at the battle of Dresden was struck by a musket ball on the outer ankle; there was only that single wound on the limb, but the ball was extracted from the middle of the thigh. Under certain circumstances a ball may be reflected at a very acute angle. Marjolin mentions a case where two officers, whilst engaged in the chase of the wild boar, quarrelled; a meeting was, in consequence, fixed for the next day. During the chace, by chance, they were both together—a boar passed, one of them fired, and his adversary fell dead. Suspicion at once fixed upon the survivor the wilful destruction of his adversary. An old military surgeon was present, and knowing the effect of hard substances upon the course of bullets, carefully examined the place, and found upon an old oak, about the height of a boar, a very hard knot which bore the mark of the bullet. A spent ball may strike the anterior surface of the thigh, and may escape posteriorly, without passing through the muscles. In the attack upon Newport, a man was shot in front of the chest; the ball was found behind, but it did not penetrate that cavity. It may be received in the forehead, and extracted from the occiput, but without penetrating the skull.

Usually, a gun-shot wound has the form of the body which produced it, whether round, square, or oblong; but when there are two openings, that by which it has entered is almost always smaller than that by which it has escaped; its borders are depressed, whilst that of escape are raised like a tumour. This happens because at the time of entering, the velocity is greatest; but this is open to numerous exceptions—the ball may not have penetrated perpendicularly to the surface, may not have lost its velocity. If a ball strike against a bone it may be changed in form, may be flattened, may be shattered, or cut into two or more pieces. In the case of Serjeant Daly, at Newport, one slug appeared to have penetrated, but it was shattered into many pieces upon the os frontis. When balls are ill cast they often contain air, or, when the lead has not been poured into the mould continuously, this may happen—a ball may break into several pieces. One portion of a ball may, therefore, pass out; another portion may remain in the part: consequently, the existence of the openings should not prevent your examining whether any portion still remain in the wound. Some authors deny this division of balls, and maintain that, in such cases, the piece must have been loaded with two balls. The shortest answer which can be given is to say, that two balls shot out of a gun never penetrate at the same point. Wherever you find that there is but one opening through which a ball passed into the body, and you find only one half the bullet, the chances are that the other half still remains, unless indeed half bullets were used.

In many cases bullets do not penetrate alone, but carry portions of dress or wadding with them; therefore it is necessary to examine carefully the clothes of the wounded person. When a ball has lost much of its velocity, it rarely makes a fair hole in the clothes, but carries a portion before it into the wound. These wounds are usually superficial, and in undressing, the ball may be extracted, with the article of dress which it has carried before it. We may, then, have a wound in which no bullet is found; it is therefore very necessary to carefully examine the clothes.

When a ball falls, at the end of its course, it is not at rest, but revolves with extreme rapidity; and if a person attempt to take hold of it, he may suffer severely.

The contusion produced by such bodies may extend to attrition: the course of the wound is livid, and this colour is owing to the disorganization of the part, and to the blood extravasated into the cellular tissue; formerly, it was supposed to depend upon the burn occasioned by the ball. In the present day we know that the temperature of a body projected by gunpowder is not raised during its course; it is not heated except it come in contact with a very hard body, against which it is flattened. These wounds are rarely very painful at the time; the sensation usually felt is a numbness—but that stupor may be so great, and extend so far, as to destroy life. They usually bleed little, especially when the divided vessels are small; hæmorrhage would seem to be prevented by the bruising of the parts and the retraction of the extremities of the vessels. When large vessels are partially divided there is sometimes hæmorrhage; when the section is complete frequently there is no hæmorrhage. If the eschar be not soon thrown off there may be no secondary hæmorrhage; and if you have an opportunity of examining the case, you will find both ends of the artery obliterated. In many wounds where a limb has been carried away near the trunk, by a cannon ball, there is no bleeding. Gun-shot wounds are rarely healed by first intention; there is attrition—there must consequently be sloughs and suppuration. No contused wounds are so dangerous as these, and this it was which induced the old surgeons to suspect poison.

A fatal wound may be inflicted, though the fire-arm contain no projectile; but then it must be fired off very near the person. A soldier wishing to destroy himself was suspected—his pistols were examined, they were loaded with ball; the balls were extracted by his comrades. He put one of the pistols in his mouth, fired it off; it burnt the tongue, blew out some of the teeth, fractured the jaw, and he died on the third day from cerebral concussion.

The wadding, if firm, may cause a mortal wound: usually it is intercepted by the dress, but now and then it penetrates to a short distance, and remains in the wound. It should be promptly extracted, or it may cause much irritation, and may remain in the part for years. In these wounds the stupor of the part and the concussion are less decided than in bullet wounds. A very brittle or soft body may equally penetrate; you may fire a candle, at thirty paces, deep into an inch board.

There is an important class of wounds about which it is necessary I should speak; those deep-seated and serious contusions sometimes produced while the integuments over the part appears uninjured; formerly supposed to be produced by the "wind of a ball." It is hardly necessary for me to lay before you the different theories upon which that opinion was based. Certainly the facts prove that the disorders observed do not result from a contusion produced by the displacement of a column of air. Many curious facts are recorded by military surgeons to demonstrate the impossibility of such injuries. The only reasonable explanation of such injuries is, that the ball has struck the integuments very obliquely; the skin yields, the deeper seated parts resist. Whatever the explanation, you must accept the fact—that the skin may be comparatively uninjured, while deeper seated parts are very seriously damaged.

Wounds caused by the bursting of fire-arms: we often see they are very variable; usually it is the left hand which suffers: the manner in which the piece is held also determines to some extent the situation of the wound—some persons grasp the barrel firmly with the hand; in most cases the injury falls between the thumb and fore-finger; it may lacerate this part, may blow off the thumb. Sometimes the superficial palmar arch is injured, and there is much hæmorrhage; at others the ends of the artery retract, and the bleeding is trifling. The surface is red, not black or livid, as when the part is struck by a bullet. If the articulations are uninjured, these wounds often do well without mutilation. Even when the thumb and indicator finger are blown off, it will often do well.

If a joint of the finger be opened, should we attempt to save it, or should we amputate at the joint, or in the continuity ?—According to the importance of the part should we anxiously seek to preserve the part. Some of these wounds will heal. If all the fingers are carried away, the thumb itself being wounded, we should endeavour to save it because of its usefulness, even though the stump be irregular. Occasionally the wrist is affected : if the wound be slight, whether penetrating the joint or not, every effort must be made to save the hand ; but if the injury to the carpal bones be great, amputation will be necessary. Dupuytren had an idea that amputation of the hand should hardly ever be performed in cases of wounds of the wrist; in this I think he is wrong—I have known lives lost in the endeavour to avoid such amputation. It is, however, very difficult to define the cases in which the operation is absolutely required.

*Symptoms.*—I have said that generally gun-shot wounds are not painful ; the hæmorrhage is usually trifling : the diameter of the opening is no exact criterion of the diameter of the projectile : the edges are of a blackish red colour; this colour depends upon the attrition of the flesh, and the infiltration of blood. The symptoms which accompany them are primary or secondary. Among the primary symptoms are hæmorrhage and concussion ; the first is usually trifling, but it may be enough to destroy life. It is the belief of many military surgeons that of those who die on a field of battle, a large number die from hæmorrhage. The concussion is a consequence of the shock inflicted upon the part ; it may extend to a considerable distance—may be very severe when a hard part is struck, such as the cranium. It may produce a state of stupor of the part, from which the patient may not recover. It was so in the case of a soldier described by Quesnay; in him the hebetude was so great that when amputation was proposed to him, he answered that it was no affair of his. This stupor is the state in which a part of the body has almost entirely lost sensation and motion. In a limb so affected, the circulation is almost or altogether arrested ; the temperature is much depressed. If you cut into a part in this state, even at some distance from the seat of injury, blood does not flow. When this state extends beyond the injured limb, it is then general. Frights will sometimes produce similar accidents ; the face is pale, discomposed, covered with cold sweat, and the power of speech is lost. In 1814 a man was picked up in this state on the field of battle who had not a scratch about him; he was senseless for more than two hours. Sometimes instead of stupor we see spasm or general tremor ; sometimes rigors : the skin is corrugated, the pulse is hard and small, the countenance pale, and covered with cold sweat. When these symptoms are owing to fright or passion, warmth and stimuli will soon dissipate them. If the concussion extend to the liver there is often icterus. Tetanus may supervene immediately upon gun-shot wounds.

The hæmorrhage most to be feared is consecutive, for then we cannot get at the vessel ; and if we could, the tunics will be so softened by inflammation that a ligature will at once cut through them. These hæmorrhages may occur from the seventh to the fifteenth day after the accident. In these cases all that remains for us is ligature at a distance, or amputation ; and it is often difficult to decide between them ; when the limb is much injured amputatiom may be preferred, because gangrene in such cases is apt to sugervene after the ligature. Amputation itself is uncertain in its results, in a patient enfeebled by pain, suppuration, and constant small hæmorrhages.—*Lond. Med. Gaz.*

---

*Lactucarium.* By Dr. Fisher, Knöig. Hofrathe und kreis-phisicus zu oels.—Aware that the list of medicines requires sifting and arrangement rather than addition, nevertheless the author of this paper desires, from ten years' experience, to obtain for Lactucarium a permanent place in the materia medica.

Lactucarium is known to be the juice obtained by making incisions in the stalk of the Lactuca sativa and virosa, and left to dry in the open air. It has an acrid taste and bitter smell, and contains malic acid, potassa, resin, and a glutinous matter, but no morphia. According to Buchner, the active principle is Lactucin, which is combined with the resin. The Luctuca virosa will yield three times as much Lactucarium as the Lactuca sativa.

The Lactucarium gallicum, (parisiense, *theridax* (Θριδαξ) *theridace*), is obtained from the Lactuca sativa; the Lactucarium anglicum, from the virosa. The latter is very much the stronger, of which, according to Rothnel, half a grain will produce the same effects as two or three grains of the French preparation.

During the first century, A. D., the seed and the juice of the stalk of the Lactuca sativa were in high repute for their sedative powers. And many modern physicians (Francois, Coxe, Rothanel, Hueter,) who restored the use of the forgotten remedy, agree as to its efficacy.

The Lactucarium quiets without previously exciting, and produces sleep without insensibility; it allays morbid sensibility, lessens the action of the heart, and produces a general tranquillity throughout the whole constitution. In large doses it produces delirium without stimulating, diminution of nervous power, disturbance of the digestive functions, and vomiting.

The Lactucarium administered in small doses confines its effects to the periphery of the nervous system; in larger doses it affects the brain, and produces sleep by relaxing and exhausting the sensibility. It is, consequently, applicable wherever it is required to allay nervous irritation, and reduce an excessive action of the vascular system originating in an abnormal state of the nervous functions, and where, on that account, antiphlogistics must be aided by sedatives and antispasmodics.

The field for its administration is in all the neuroses and neuralgia, especially the convulsions of childhood, when they do not originate in stomach affections, convulsions during difficult parturition (Hüter wendt Rothamel, Wiesner) spasmodic asthma, hooping cough, and cramp in the stomach. Material relief is afforded by Lactucarium to the distress and want of sleep attending hydrothorax and diseases of the lungs, as well as to the restlessness and anxiety of hysteria. Hyeter recommends it in spasmodic hæmorrhages, (spastichen blutungen) irregular hæmorrhoids, &c. It might, perhaps, be administered in the second stage of the arachnitis cerebralis and spinalis of children, when the bleedings, cold affusions, &c., which have been used for the tonic and clonic convulsions, have been of no avail, and we dare not give narcotics on account of the irritable state of the patients. It is probably useful in mania by diminishing the increased irritability of the brain, when we are afraid to continue the powerful antiphlogistics and derivative drain upon the alimentary canal, lest they should produce excessive debility and imbecility. Therefore Bramer rightly considers the Lactucarium as a medicine which, in delirium tremens, allays the excited state of the cerebral system, and thus induces the critical sleep, without the previous and simultaneous excitement of the vascular system, which is caused by opium and hyoscyamus.

To determine the question of whether the Lactucarium should be retained in the materia medica or not, we should inquire whether any and what peculiarities are discoverable in the juice of this plant, by a comparison of its effects with the effects of the narcotic substances which are now generally used as medicines.

From *opium* it differs in that it will bear no comparison with it as regards its narcotic effects; and likewise that it does not restore (as opium does) the normal relation to each other of the two agents of organic life by previously increasing the action of the vascular system, and therefore it is not contraindicated (as opium is) by a morbid excitement of the circulation.

From *belladonna* it differs in that the belladonna blunts, and at length destroys the sensibility of the nerves, whereas the sedative effects of the Lactucarium are the primary, and its action mild; it may, therefore, be administered in morbid sensibility which has nearly exhausted itself, and in inflammations, where the morbid irritability has passed into a state of constant pain: whereas belladonna can only be serviceable by destroying the sensibility.

The *conium maculatum*, like all narcotics, depresses the nervous system, but with this peculiarity, that it lessens arterial and muscular action by increasing the action of the veins. (?) The Lactucarium allays the sensibility immediately, without any effect upon the nervous system.

*Hyoscyamus* is a narcotic which produces mild but purely sedative effects: it brings on a state of paralysis as the consequence of a primary excitement of the nerves, so that the fatal narcotic effects of hyoscyamus are produced, not by apoplexia cerebralis sanguinea, but by paralysis of the nerves. It differs, therefore, from the Lactucarium by the sedative effects of the Lactucarium being obtained by a direct lowering of the sensibility, whereas these effects of hyoscyamus are preceded by an irritation of the nervous system.

Lactucarium is, therefore, recognised as a medicine possessing peculiar therapeutic properties, inasmuch as it quiets the nervous system, and, although given in moderate doses, it cannot allay morbid sensibility of the nerves in a higher or even an equal degree with the pure narcotic, and yet it produces its sedative effects without being attended or followed by other effects which limit or altogether forbid the employment of such substances.

The Lactucarium is properly a sedative and paregoric, a link between hydrocyanic acid and hyoscyamus, and, therefore, since it is neither more costly nor more nauseous than other narcotics, it may justly lay claim to a place in the materia medica. It is liable to spoil by keeping, and, from being insoluble in water, it forms a sediment in mixtures, which are the only objections to it.—*Lond. Med. Gaz., from Rust's Magazine*, 53 Band. 1 Heft.

---

*On the Prevention of Tubercles.*—In a letter addressed to the Royal Academy of Medicine, M. Coster announces that, from certain experiments which he has made, he hopes to prove,

1. That it is possible, even in the face of predisposing causes, to prevent the development of the tubercular diathesis.

2. That even where the formation of tubercles has commenced, their progress may, in a great number of cases, be arrested.

The following are a few of the experiments upon which M. Coster has built up his hopes:

Two years ago he placed a number of dogs, rabbits, &c., in the circumstances most favourable to the development of the scrofulous diathesis. Thus, many of the unfortunate animals were shut up in dungeons, without light, incapable of moving, and exposed to a moist cold by means of wet sponges which were hung up in the cages. Some of the animals placed in these conditions, were fed on their ordinary diet; others were fed with ferruginous bread containing ½ oz. of carbonate of iron to the pound. All the former became ill, the greater part tuberculous, but not one of those fed on the bread containing iron presented a trace of tubercles.—*London Lancet, from Bull. de l'Acad.*, Jan. 31, 1840.

